# Correction to: Effects of early extubation followed by noninvasive ventilation versus standard extubation on the duration of invasive mechanical ventilation in hypoxemic non-hypercapnic patients: a systematic review and individual patient data meta-analysis of randomized controlled trials

**DOI:** 10.1186/s13054-021-03692-5

**Published:** 2021-08-03

**Authors:** Rosanna Vaschetto, Alessandro Pecere, Gavin D. Perkins, Dipesh Mistry, Gianmaria Cammarota, Federico Longhini, Miguel Ferrer, Renata Pletsch-Assunção, Michele Carron, Francesca Moretto, Haibo Qiu, Francesco Della Corte, Francesco Barone-Adesi, Paolo Navalesi

**Affiliations:** 1 Azienda Ospedaliero Universitaria “Maggiore della Carità”, Anestesia e Terapia Intensiva, Novara, Italy; 2 Dipartimento di Medicina Traslazionale, Università del Piemonte Orientale, via Solaroli 17, 28100 Novara, Italy; 3 Warwick Clinical Trials Unit, Warwick Medical School, Warwick University, Gibbet Hill, Coventry, UK; 4 Anestesia E Rianimazione, Dipartimento Di Scienze Mediche E Chirurgiche, Università “Magna Graecia”, Catanzaro, Italy; 5 RIICU, Department of Pneumology, Respiratory Institute, Hospital Clinic of Barcelona, IDIBAPS, CibeRes (CB/06/06/0028), University of Barcelona, Barcelona, Spain; 6 Department of Physiotherapy, Centro Universitário Padre Anchieta, UNIANCHIETA, Jundiaí, SP Brazil; 7 Department of Medicine - DIMED, Section of Anesthesiology and Intensive Care, University of Padua, Padua, Italy; 8 Department of Critical Care Medicine, Zhongda Hospital, School of Medicine, Southeast University, Nanjing, 210009 Jiangsu China

## Correction to: Vaschetto Crit Care (2021) 25:189 10.1186/s13054-021-03595-5

Following publication of the original article [1], the authors identified an error in Table 1. The correct Table is given hereafter.

**Table 1:** Characteristics of the randomized control trials included in qualitative synthesis

Study	Setting	Primary endpoint	Secondary endpoints	Number of patients included in the original paper	Baseline characteristics of patients at entry into the study	Number of excluded patients and reasons	Number of patients potentially to be analyzed	Number of patients analyzed
Ferrer et al. 2003	2 Spanish hospitals	The decrease of the duration of invasive ventilation defined as positive pressure ventilation delivered through orotracheal intubation or tracheotomy, in the NIV group.	1. Total period of ventilatory support 2. ICU length of stay 3. Hospital length of stay 4. Reintubation 5. Main causes of reintubation -Severe persistent hypoxemia -Severe dyspnoea -Inability to manage secretions -Hemodynamic instability 6. Tracheotomy 7. ICU survival 8. Causes of death within 90d after entry in the study -Septic shock/MOF -Refractory hypoxemia -Cardiac arrest -Pneumothorax -Stroke -Pulmonary embolism	43 patients 21 NIV 22 Control	1. Age 2. Sex 3. Current or former smoker 4. Current of former alcohol abuse 5. APACHE II 6. Duration of ICU stay 7. Duration of mechanical ventilation 8. Number of comorbidities per patient 9. White blood cells 10. Haematocrit 11. Patients with chronic pulmonary disorders 12. Causes of mechanical ventilation -Exacerbation of chronic pulmonary disorders -Congestive heart failure -Community-acquired pneumonia -Hospital-acquired pneumonia -Postoperative respiratory failure -Acute lung injury -Thoracic trauma -Haemoptysis -Cardiac arrest	17 acute-on-chronic exacerbation COPD 9 acute cardiogenic pulmonary oedema 3 severe asthma 8 chronic pulmonary disorder	6 patients 4 Intervention 2 Control	6 patients 4 Intervention 2 Control
Trevisan et al. 2008	Single-centre Brazil	To evaluate the use of bi-level NIV for patients who fail weaning from i-MV	1. ICU length of stay 2. Hospital length of stay 3. otal length of stay in hospital 4. ICU death 5. Ward death 6. Mechanical ventilation time after randomization 7. Total mechanical ventilation time 8. Complications -Pneumonia -Sepsis -Congestive heart failure -Tracheotomy -Return to IMV -Skin necrosis	65 patients 28 NIV 37 Control	1. Age 2. Sex 3. APACHE-II 4. Duration of mechanical ventilation 5. Causes of mechanical ventilation -COPD aggravation and -Asthma -Heart diseases -Respiratory diseases -Post-surgery respiratory failure -Acute pulmonary lesion -Pneumonia -Tuberculosis -Thoracic trauma	23 acute-on-chronic exacerbation COPD and asthma 11 acute cardiogenic pulmonary oedema 5 PaCO_2_ >50 mmHg and pH >7.35 2 age <18 years old	24 patients 10 Intervention 14 Control	24 patients 10 Intervention 14 Control
Vaschetto et al. 2012	Single-centre Italy	Duration of i-MV	1. ICU length of stay 2. ICU mortality 3. Hospital mortality 4. Extubation failure 5. i-MV before T0 6. i-MV AFTER T0 7. 28-i-MV free days 8. 28-MV free days 9. Weaning 10. Side effects/complications of i-MV -Tracheotomy -Continuous i.v. sedation	20 patients 10 NIV 10 Control	1. Age 2. Sex 3. APACHE II 4. Causes of mechanical ventilation -Pancreatitis -Pneumonia -Thoracic trauma -Bowel obstruction	None	20 patients 10 Intervention 10 Control	20 patients 10 Intervention 10 Control
Carron et al. 2014	Single-centre Italy	Weaning success/failure rate	1. Duration of i-MV 2. Duration of ventilator support for weaning 3. Duration of total ventilator support 4. Weaning failure 5. Reintubation -Refractory hypoxemia -Bronchial hypersecretion -Transient ischemic attack -Hypercapnia 6. Conventional weaning after reintubation with/without percutaneous dilatational tracheostomy 7. Main complication after entry in the study -VAP -Catheter-related pneumonia -Septic shock -Multiple-organ Failure -Disseminated intravascular coagulation -Cardiogenic shock -Cardiac arrest 8. ICU length of stay 9. Hospital length of stay 10. ICU survival 11. Hospital survival	64 patients 32 NIV 32 Control	1. Age 2. Sex 3. Weight 4. APACHE II 5. ARF hypoxemic hypercapnic (n. of patients) -Exacerbation of chronic pulmonary disease -Asthma -Community-acquired bronchopneumonia -Hospital acquired-bronchopneumonia 6. ARF hypoxemic (n. of patients) -Postoperative respiratory failure -Community-acquired bronchopneumonia -Hospital acquired-bronchopneumonia -Acute cardiogenic pulmonary oedema -Congestive heart failure -Acute pulmonary embolism -Acute pancreatitis -Acute lung injury following ab ingestis -Thoracic trauma -Burn	17 acute-on-chronic exacerbation COPD 1 Asthma 5 acute cardiogenic pulmonary oedema 4 BMI ≥30 10 PaCO_2_ >50 mmHg and Ph >7.35	27 patients 14 Intervention 13 Control	27 patients 14 Intervention 13 Control
Perkins et al. 2018	41 hospitals UK	Time from randomization to successful liberation from all forms of mechanical ventilation	1. Mortality at 30, 90, 180 days 2. Duration of i-MV 3. Duration of total ventilation 4. Time to meeting ICU discharge criteria (defines as no further requirement for level 2/3 care) 5. Reintubation rates 6. Tracheostomy 7. Adverse events and serious adverse events	364 patients 182 NIV 182 Control	1. Age 2. Sex 3. Evidence of delirium 4. Body mass index 5. Duration of ventilation prior to randomization 6. Antibiotics for respiratory 7. Infections 8. APACHE II 9. Admission diagnosis -Pneumonia/respiratory infection -Post-surgery respiratory failure -Cardiac -Non-respiratory infection -Neuromuscular -COPD/asthma exacerbation -Traumatic injuries -GIT bleeding -Pancreatitis -Stroke	15 neuromuscular patients 14 COPD/asthma exacerbation 33 acute cardiogenic pulmonary oedema 48 PaCO_2_ >50 mmHg and pH >7.35	254 patients 130 Intervention 124 Control	254 patients 130 Intervention 124 Control
Vaschetto et al. 2019	6 hospitals China 3 hospitals Italy	1. Days of i-MV Overall Medical Surgical 2. ICU length of stay Overall Medical Surgical	1. Treatment failure 2. Severe events 3. Tracheostomy 4. VAT 5. VAP 6. Use of sedatives 7. Hospital length of stay 8. ICU mortality 9. Hospital mortality	130 patients 65 NIV 65 Control	1. Main causes of i-MV -ARDS -Pneumonia -Septic shock -Polytrauma -Postoperative abdominal surgery -Postoperative vascular surgery -Postoperative thoracic surgery -GIT bleeding -Cerebral bleeding -Pancreatitis 2. Days of i-MV pre-protocol 3. Days of NIV pre-protocol	2 PaCO_2_ >50 mmHg and pH >7.35	128 patients 65 Intervention 63 Control	128 patients 65 Intervention 63 Control

APACHE II, Acute Physiology and Chronic Health Disease Classification System II; ARDS, Acute Respiratory Distress Syndrome; ARF, Acute Respiratory Failure; BMI, Body Mass Index; COPD, Chronic Obstructive Pulmonary Disease; GIT, Gastrointestinal; ICU, Intensive Care Unit; i-MV, invasive Mechanical Ventilation; i.v., intravenous; LOS, Length Of Stay; MOF, Multiple Organ Failure; N.A., Not Applicable; NIV, Non-Invasive Ventilation; PaCO_2_, arterial partial pressure of carbon dioxide; PE, Pulmonary Embolism; UK, United Kingdom; VAP, Ventilator Associated Pneumonia; VAT, Ventilator Associated Tracheobronchitis

All the changes that were requested are implemented in this correction and the original article [1] has been corrected.

## References

[1] Vaschetto R, Pecere A, Perkins GD (2021). Effects of early extubation followed by noninvasive ventilation versus standard extubation on the duration of invasive mechanical ventilation in hypoxemic non-hypercapnic patients: a systematic review and individual patient data meta-analysis of randomized controlled trials. Crit Care.

